# The effect of tooth loss on the temporomandibular joint space: A CBCT study

**DOI:** 10.1002/cre2.845

**Published:** 2024-01-30

**Authors:** Salma Tabatabaei, Maryam Paknahad, Mahdi Poostforoosh

**Affiliations:** ^1^ Oral and Dental Disease Research Center, Department of Oral and Maxillofacial Radiology, School of Dentistry Shiraz University of Medical Sciences Shiraz Iran; ^2^ Department of Pediatric Dentistry, School of Dentistry Kerman University of Medical Sciences Kerman Iran

**Keywords:** cone‐beam computed tomography, Eichner index, mandibular condyle, temporomandibular joint spaces

## Abstract

**Objective:**

The tooth loss has a significant impact on the positioning of the condyle in the glenoid fossa and joint spaces of the temporomandibular joint (TMJ). The aim of this study was to assess the association between tooth loss and TMJ spaces using cone beam computed tomography (CBCT) images.

**Materials and Methods:**

This retrospective investigation involved the evaluation of CBCT images of the bilateral TMJs in a cohort of 111 individuals, comprising 68 males and 43 women. The dentition of the patients was categorized into three categories, including A (65.4%), B (19.1%), and C (16.4%), based on the Eichner index. Anterior, superior, and posterior joint spaces were then measured in sagittal views. The Kruskal–Wallis test and Mann–Whitney test were employed to identify significant differences among the three Eichner groups.

**Results:**

The findings of the present study suggested that there was no statistically significant variation in the anterior joint space among different Eichner groups within the general population (*p* = .781). Nevertheless, the superior and posterior joint spaces exhibited statistically significant alterations, as indicated by *p*‐values of .039 and .010, respectively. It was detected that condyles were positioned inferiorly and posteriorly in group C when compared to groups A and B.

**Conclusion:**

The present study indicated that greater loss of tooth‐supporting zones is associated with posterior and inferior displacement of condyles. Understanding these relationships helps emphasize how crucial it is to replace missing teeth to enhance occlusion support and, in turn, stop the progression and further deterioration of temporomandibular disorders.

## 1 INTRODUCTION

The temporomandibular joint (TMJ) is a multifaceted synovial articulation that connects the mandibular condyle to the glenoid fossa and articular eminence of the temporal bone (Whyte et al., 2021). The radiographic joint space refers to the radiolucent region that exists between the mandibular condyle and the temporal bone (Paknahad, Shahidi, Iranpour, et al., 2015). The clinical importance of joint space holds considerable significance since the existence of a proper joint space is essential for facilitating unrestricted movement of the condyle in conjunction with the articular disc (Mallya, 2019). Changes in condylar position over time can lead to structural modifications of the TMJ surfaces, triggering symptoms of temporomandibular joint disorders (TMD), such as discomfort and dysfunction (Ammanna, 2009).

The interrelationship between the anatomical and physiological aspects of the TMJ is closely associated with each constituent of the masticatory apparatus (Ammanna, 2009). The teeth have a crucial role in establishing a secure vertical and distal relationship between the mandible and maxilla. Additionally, they serve as guiding planes for the anterior and lateral movement of the jaw throughout the range of motion where the teeth are in contact (Shehab et al., 2011).

The Eichner index, which was designed by Karl Eichner, is a commonly used dental index that finds application in epidemiological research within the field (Hiltunen et al., 2002; Paknahad, Khojastepour, et al., 2023). This index has been found to be beneficial in establishing intermaxillary connections and extending functional dental invalidity. According to this index, the posterior teeth can be categorized into four support zones, which are determined by the presence or absence of occlusal contact between the premolars and molars (Paknahad, Khojastepour, et al., 2023; Paknahad et al., 2002).

Several studies have shown that tooth loss can greatly affect the TMJ morphology in several ways. The absence of posterior teeth, in particular, and the subsequent loss of the occlusal curve can disrupt the balance between adaptation and functional harmony, leading to impaired functionality (Shehab et al., 2011). Furthermore, the loss of teeth might have an impact on the inclination of the articular eminence through the process of remodeling (Shehab et al., 2011).

Regarding the impact of tooth loss on the TMJ spaces, it has been shown that the reduction in vertical facial height due to tooth wear and tooth loss might lead to the posterior and superior displacement of the condyles inside the joint space (Hongchen et al., 1992). According to a study conducted by Yanikoglu and Guldag (2006), it was found that patients with bilateral loss of posterior tooth‐supporting regions have a reduction in the anterior, superior, and posterior joint spaces, as compared to patients with unilateral posterior tooth‐supporting area. Similarly, a study conducted by Amorim et al. (2003). revealed a significant reduction in the posterior joint space following the loss of posterior teeth. Despite the observed alterations in TMJ joint spaces in previous studies, in a study conducted by Arıkan et al. (2022) evaluating the anterior, superior, and posterior joint spaces in dentate individuals compared to edentulous patients, no statistically significant differences were identified in any of the evaluated joint spaces. The presence of debates in this area presents a challenge in establishing a definitive conclusion regarding the impact of tooth loss on alteration in joint space within the TMJ. Radiographic examination is an integral component of the clinical examination in patients with TMDs. Several imaging modalities have been utilized to visualize the TMJ, such as plain film radiography, conventional tomography (CT), computed tomography, cone‐beam tomography, and magnetic resonance imaging (MRI). Cone beam computed tomography (CBCT) is a developing technique that is being progressively more used in dentomaxillofacial imaging due to its relatively low‐dose, high‐spatial‐resolution characteristics (D'Angeli et al., 2020; Sırlı Yılmazturk et al., 2023). This modality provides accurate and reliable linear measurements for imaging of dental and maxillofacial structures. These measurements are extremely beneficial in clinical practice when treating patients with TMD. Hence, CBCT is the modality of choice for the assessment of temporomandibular osseous structures (Paknahad & Shahidi, 2015; Paknahad et al., 2016).

Due to ongoing controversy as well as the use of two‐dimensional radiography as a main modality of choice for image analysis in the majority of previous studies, the principal objective of this study attempt was to investigate the potential correlation between tooth loss and the TMJ space using CBCT images.

## 2 MATERIALS AND METHODS

The current investigation received approval from the Institutional Research Committee under the reference number IR.SUMS.DENTAL.REC.1402.004. The CBCT images of 111 patients were analyzed in this study, 68 (61.8%) females and 43 (38.2%). It's worth noting that the images were originally taken for different purposes. The patients' ages ranged from 19 to 83 years old (45.73 ± 16.93). Patients with a history of temporomandibular surgery, acute trauma, severe skeletal malocclusion, congenital abnormalities, musculoskeletal, or neurological disorders, and any systemic diseases that could affect joint morphology, such as rheumatoid arthritis, were all excluded from the study. All participants signed an informed consent that their anemone data may be used in further research.

### 2.1 Tooth loss recordings

**Figure 1:**
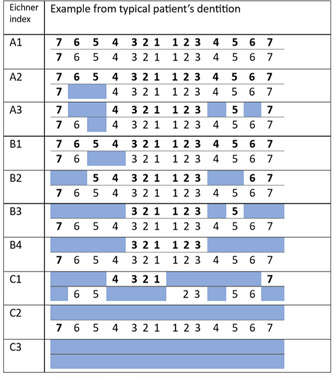
Classifications of Eichner index.

The patient's dentition was categorized into four primary occlusal supporting zones based on the occluding pairs seen in the posterior teeth (two premolars and two molars). In class A, there is contact among all four supporting zones. In class B, one supporting zone is absent, or all four supporting zones are lost, but the anterior region remains intact. Class C is characterized by the absence of occlusal contact between the remaining teeth (Figure 1). This study classified both fully and partially erupted permanent teeth as “present teeth.” Furthermore, it should be noted that the supernumerary teeth, third molars, pontics of bridge prostheses, and implant‐supported superstructures were not counted as present teeth.

### 2.2 CBCT of TMJs

The bilateral TMJ CBCT images were taken using a New Tom VGi (NewTom) imaging equipment. Imaging settings included 110 KVp voltage peak, 3.05 mA current, and 3.6 s exposure. The images were taken in standard resolution mode (0.3 voxels). The imaging fields were 15 × 15 cm, with patients standing erect; patients were asked to bite down in their maximal intercuspal position. The Frankfort plane maintained parallel alignment with the floor when their heads were positioned. The NewTom Cone Beam 3D imaging system workstation (NNT Software version 6.2) was used to process TMJ images. The evaluation of the CBCT scans was conducted using a high‐resolution Barco‐China monitor in a specialized reporting room with proper viewing conditions, including a dimly illuminated environment.

### 2.3 Joint space measurement

**Figure 2:**
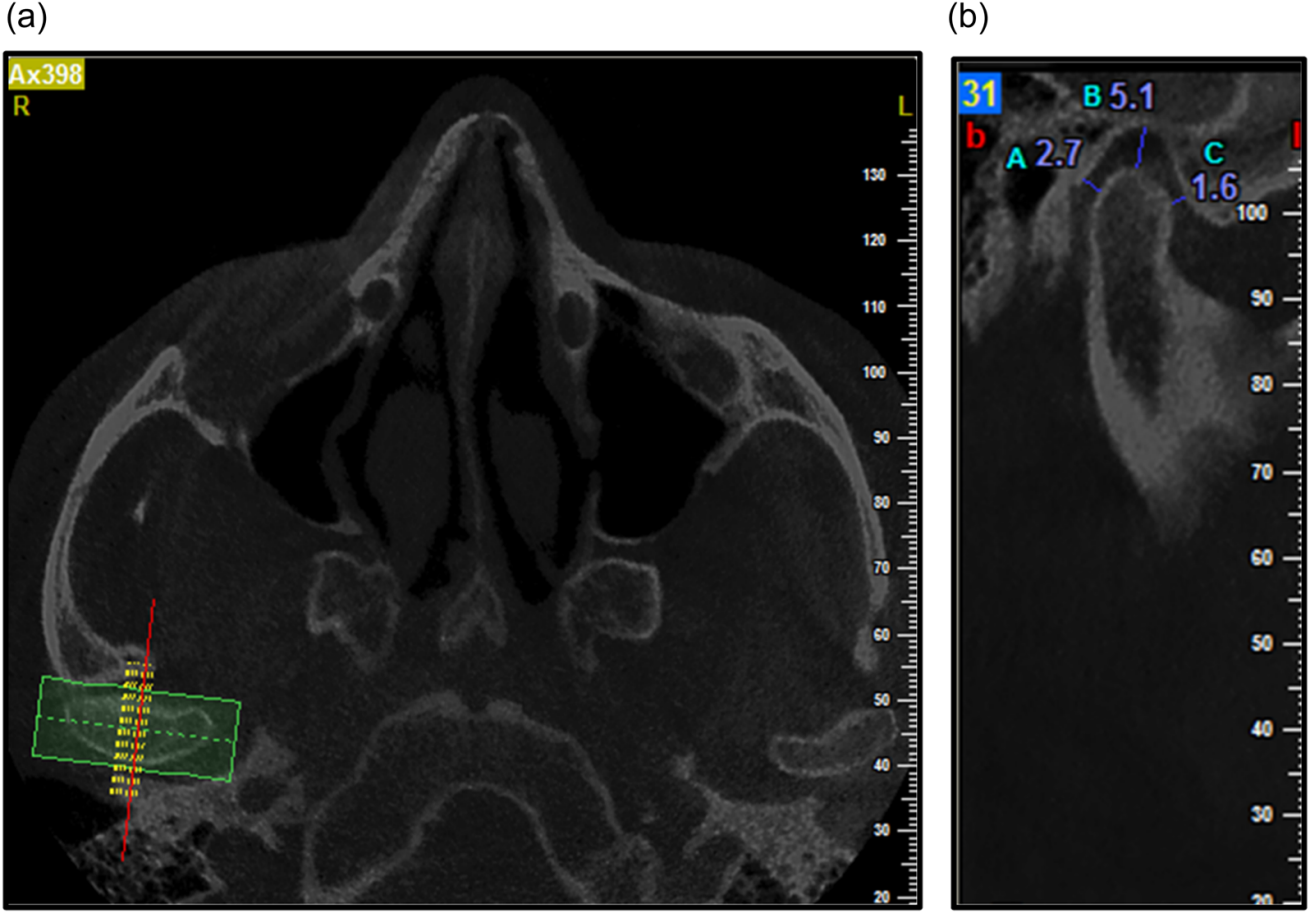
Linear measurement of posterior (A), superior (s), and anterior (C) subjective closest joint spaces in a sample patient. (a) Axial view and (b) corrected sagittal view.

The focus of the data reconstruction was on the TMJ. The axial view displaying the condylar process with the greatest mediolateral diameter was selected for secondary reconstruction. A line parallel to the longitudinal axis of the condylar process was drawn on this image. Corrected sagittal slices were then reconstructed using a slice interval and thickness of 0.5 mm. On the central sagittal section, the narrowest posterior (P), anterior (A), and superior joint space values were assessed accurately on both the left and right sides. All CBCT images were measured by an oral and maxillofacial radiologist (Figure 2).

### 2.4 Statistical analyses

Statistical calculations were performed using SPSS (version 25). To assess the presence of significant observer errors during measurement, the same operator remeasured all CBCT images 2 weeks later. The intraclass correlation coefficient (ICC) was utilized to evaluate the congruence between the initial and subsequent assessments. To evaluate the interoperator method error, a random sample comprising roughly 30% of the acquired data was chosen and afterward remeasured by another oral and maxillofacial radiologist. The final data were subsequently subjected to comparison through the utilization of ICCs. The Kruskal−Wallis test and Mann−Whitney test were employed to identify significant differences regarding joint spaces among the three groups. The *χ* ^2^ test was used to determine whether there was a significant association between joint spaces and Eichner groups, considering different gender and age groups. The significance level was set at *p* = .05.

## 3 RESULTS

The resulting inter‐class correlation coefficient (ICC) value of 0.967 indicates a substantial level of agreement between the initial and subsequent measurements, thereby validating the reliability of the measures. Furthermore, a notable degree of agreement was noted among the operators for all measurements (ICC ≥ 0.90).

The result of this study showed that the anterior joint space was not significantly different between the three groups. However, superior and posterior joint spaces were significantly different between the three groups. It was shown that condyles were posteriorly and inferiorly positioned in group C and compared to group A (Table 1).

**Table 1:** Descriptive statistics and comparison of variables among three Eichner classifications.

	Eichner groups			
Joint space	A	B	C	*p* Value
Anterior	1.9 (1.6 ± 2.5)	2 (1.6 ± 2.65)	1.95 (1.5 ± 2.77)	.781
Superior	2.4 (2 ± 2.8)^c^	2.5 (2.25 ± 3.32)	2.9 (1.9 ± 3.27)	.039*
Posterior	2.45 (1.42 ± 3)^C^	2.1 (1.5 ± 3.2)	1.7 (1.5 ± 2.3)	.010*

*Note*: Post hoc: A and C → superior joint space → *p* = .033, A and C → posterior joint space → *p* = .026.

* Significant.

There was a significant association between condylar position and Eichner index in male and female patients. In female patients, the condyles tended to be located more posteriorly and inferiorly in group C than in group A (Table 2).

**Table 2:** Descriptive statistics and comparison of variables among three Eichner classifications in female patients.

	Eichner groups			
Joint space	A	B	C	*p* Value
Anterior	1.7 (1.5 ± 2.1)	1.9 (1.5 ± 2.5)	1.8 (1.5 ± 2.9)	.672
Superior	1.8 (1.9 ± 2.8)^c^	1.9 (1.5 ± 2.57)	2.7 (2.22 ± 3.52)	.014*
Posterior	2.7 (1.6 ± 3.77)^c^	1.7 (1.5 ± 2.3)	1.6 (1.22 ± 2.85)	.047*

*Note*: Post hoc: A and C → superior joint space → *p* = .011, A and C → posterior joint space → *p* = .043.

* Significant.

In male patients, the condyles were positioned more posteriorly in group C compared to group A (Table 3).

**Table 3:** Descriptive statistics and comparison of variables among three Eichner classifications in male patients.

	Eichner groups			
Joint space	A	B	C	*p* Value
Anterior	1.7 (1.5 ± 2.27)	2.6 (2.32 ± 3.10)	2 (1.62 ± 2.37)	.318
Superior	2.15 (1.72 ± 2.8)	3 (2.22 × 3.30)	1.9 (1.5 ± 2.7)	.259
Posterior	2.7 (1.85 ± 3.07)^c^	2.6 (2.32 ± 3.4)	2.05 (1.22 ± 2.67)	.024*

Post hoc: A and c → posterior joint space → *p* = .024.

* Significant.

Based on different age groups, a significant association was found between Eichner groups and joint space in adult and elderly patients. In adult patients, the condyle tends to be positioned more inferiorly in Eichner group C compared to group A. In elderly patients, the condyle was located more posteriorly in group C than in group B (Table 4).

**Table 4:** Descriptive statistics and comparison of variables among three Eichner classifications in different age groups.

		Eichner groups			
Age group	Joint space	A	B	C	*p* Value
Young adult (18−35)	Anterior	1.9 (1.52 ± 2.5)	1.3 (1.1 ± 0.00)	0	.079
	Superior	2.4 (1.9 ± 2.7)	2.25 (1.9 ± 0.00)	0	.887
	Posterior	1.7 (1.35 ± 2.10)	1.5 (0.9 ± 0.00)	0	.645
Adult (35−65)	Anterior	1.9 (1.6 ± 2.4)	2 (1.6 ± 2.45)	2.25 (1.42 ± 3.52)	.604
	Superior	2.1 (2.12 ± 3.00)^c^	2.20 (1.5 ± 2.7)	3 (2.4 ± 3.43)	.029*
	Posterior	1.7 (1.52 ± 2.37)	2.15 (1.5 ± 3.2)	2 (1.27 ± 2.35)	.344
Elderly (>65)	Anterior	1.85 (1.70 ± 3.82)	2.7 (1.47 ± 3.12)	3.10 (2.8 ± 0.00)	.0490
	Superior	3.75 (3.6 ± 0.00)	2.75 (1.45 ± 3.25)	2.62 (1.97 ± 3.32)	.164
	Posterior	3 (3.0 ± 3.0)^c^	2.45 (1.65 ± 3.17)	1.6 (1.5 ± 2.62)	.034*

Post hoc: A and c (adult group) → superior joint space → *p* = .031, B and C (elderly group) → anterior joint space *p* = .04.

* Significant.

## 4 DISCUSSION

It is imperative to recognize that the analysis of condylar positioning in clinical settings holds significant importance in identifying factors that may increase the likelihood of TMJ disorders in the future (Abdel‐Fattah, 1989; Crawford 1999; Padmanabhan et al., 2012). The findings of our investigation revealed a correlation between the extent of tooth loss and the displacement of the condylar head within the glenoid fossa. When compared to fully dentate patients, it was discovered that condyles were located more posteriorly and inferiorly in group C in both genders.

Consistent with our findings indicating a reduction in the posterior joint space among edentulous individuals, a study was conducted by Amorim et al. (2003); it revealed that the absence of posterior dental support can lead to a decrease in the posterior joint space. Additionally, it has been postulated that this phenomenon may increase the likelihood of anterior disk displacement and subsequent TMD. This finding is additionally supported by Yanikoglu and Guldag (2006), who have also provided evidence that individuals with Class I Kennedy classification (characterized by bilateral loss of posterior tooth support) exhibit significantly smaller values in the posterior joint space compared to individuals with Class II Kennedy classification (characterized by unilateral loss of posterior tooth support). In contrast to our research, Arikan et al. (2022) conducted a study in which they found no significant impact on the posterior joint space due to the presence or absence of teeth. Despite the utilization of the CBCT technology in their investigation and the similarity in measuring techniques with the current study, the observed dissimilarity can be attributed to morphological changes that may arise from ethnic differences across the studies.

Regarding the inferior displacement of the condyle in edentulous individuals compared to dentate participants, previous studies have yielded conflicting outcomes. According to the research conducted by Yanikoglu and Guldag (2006), it was observed that the superior joint space in individuals with Kennedy Class I is comparatively less than those with Kennedy Class II. This finding was further corroborated in the study conducted by Rokni and Ismail (1979), wherein it was revealed that the superior joint space in edentulous patients is comparatively smaller than that observed in dentate individuals. Yanikoglu and Guldag (2006) have explained that as a result of tooth loss, the condylar head tends to move backward and upward, which would decrease the superior joint space. In contrast to the aforementioned findings, Arikan et al. (2022) have demonstrated that there is no statistically significant disparity in superior joint space between edentulous patients and dentate persons. However, upon evaluating the mean values in the study conducted by Arikan et al. (2022), it was observed that the mean values of the superior joint space were comparatively higher in edentulous patients in comparison to dentate persons. The discrepancy between the findings of the aforementioned research and the current investigation may be ascribed to variations in the duration of tooth loss experienced by the patients. Over an extended duration, there is an increased likelihood of morphological remodeling occurring in the skeletal structures of the TMJ. According to Rosado et al. (2021), there is a notable decrease in bone volume in the glenoid fossa among those who are edentulous. Furthermore, a further investigation conducted by Chen et al. (2022) revealed that the loss of teeth, even unilaterally, can lead to a reduction in condyle bone volume in an in vivo experimental model. Hence, the duration of edentulism may influence the morphological alterations of the condylar process and, consequently, its positioning within the TMJ.

Concerning the impact of age on the measured joint spaces in different Eichner groups, it was found that young adults (18−35) did not exhibit any difference among the Eichner groups when analyzing the joint spaces. It should be pointed out that group C of the Eichner classification did not exist in this age group. On the other hand, the adult patients (35−65) only showed significant changes in the quantities of the superior joint space, which was lowest in group A patients, followed by group B and group C. In the analysis of the elderly patients (<65), it was found that only the posterior joint space values indicated a statistically significant difference between the Eichner groups. Posterior joint space was found to be significantly higher in group C compared to groups A and B. Based on the theoretical speculations, it is hypothesized that the most affected parameters in the elderly (<65) patients are the posterior joint space values due to the higher degree of the loss of vertical dimension in these patients.

In the study by Yanikoglu and Guldag (2006), it was revealed that the age of edentulous patients was only associated with an anteroposterior position of the condyle, which would further impact the anterior and posterior joint spaces. This finding aligns with our research findings about the statistical significance of the posterior joint space in elderly individuals.

To assess various specific variables in the TMJ space, several radiographic modalities have been used, including plain film radiography (Gharge, Ashwinirani, & Sande, 2020; Jalalian & Alaei, 2020), CT (Gupta et al., 2020), computed tomography (Meng et al., 2021; Song et al. 2020), CBCT (Bertram et al., 2018; Shahidi et al., 2018), and MRI (Ertem et al., 2020; Mazza et al., 2020). Since conventional radiography, such as panoramic radiography, has the limitation of converting a 3D space into a 2D image, calculating the exact values of joint space could be really challenging and even unreliable. Besides, the 1−3 mm thickness of slices limits the accuracy of careful examination of condylar morphology. On the other hand, MRI could be a useful approach when assessing soft tissue with limited applicability in hard tissue evaluation. In this regard, CT and CBCT are considered the most effective methods of assessing the joint spaces, while CBCT holds the advantage of submillimeter spatial resolution, which makes it an ideal approach for high spatial resolutions (Paknahad & Shahidi, 2015).

In the present study, the pontics of bridge prostheses and implant‐supported superstructures were not counted as present teeth. The Eichner index classifies the intermaxillary connections of the posterior teeth based on the “naturally existing teeth.” This method of classification has been considered in many previous studies (Esfehani et al., 2022; Paknahad, Barzegar, et al., 2023; Paknahad, Khojastepour, et al., 2023; Yoshino et al., 2012). Few studies considered fixed prostheses in the premolar and molar regions as the constitution for occlusal support (Mathew et al., 2011; Sakakibara et al., 2014). However, the results can be altered by the presence of fixed implant‐supported restorations in the posterior jaws. Therefore, the first method of classification was chosen in this study.

The objective of this study was to investigate the potential correlation between TMJ spaces and the Eichner index. However, it is important to note that certain variables, such as the duration between tooth extraction and imaging, as well as oral habits, were not taken into consideration throughout the evaluation process. Furthermore, increasing the sample size and incorporating the Eichner subclassification of patients could enhance the study's quality and facilitate the identification of potential subtle correlations. To validate or challenge the findings of the current study, it is imperative to undertake more research while considering these criteria.

## 5 CONCLUSION

The results of this study suggested that the anterior joint space in TMJ is not associated with the degree of patients' tooth loss; nevertheless, posterior and superior joint spaces are directly associated with the degree of posterior tooth loss. These modifications have the potential to cause disturbance in the harmony of TMJ and the stomatognathic system. Hence, by recognizing these impacts, it becomes increasingly viable to preserve the equilibrium in the TMJ and stomatognathic system by prompt restoration of the missing teeth.

## AUTHOR CONTRIBUTIONS

*Study conception and design*: Maryam Paknahad. *Data collection*: Mahdi Poosforoosh. *Analysis and interpretation of results*: Maryam Paknahad. *Draft manuscript preparation*: Maryam Paknahad. All authors reviewed the results and approved the final version of the manuscript.

## CONFLICT OF INTEREST STATEMENT

The authors declare no conflict of interest.

## Data Availability

Data that support the findings of this study are available from the corresponding author upon reasonable request.
